# Urinary Peptide Profiling to Differentiate between Minimal Change Disease and Focal Segmental Glomerulosclerosis

**DOI:** 10.1371/journal.pone.0087731

**Published:** 2014-01-30

**Authors:** Vanessa Pérez, Meritxell Ibernón, Dolores López, María Cruz Pastor, Maruja Navarro, Maribel Navarro-Muñoz, Josep Bonet, Ramón Romero

**Affiliations:** 1 Department of Nephrology, Hospital Universitari Germans Trias i Pujol, Universitat Autònoma de Barcelona, Esfera UAB, Badalona, Spain; 2 Laboratory of Experimental Nephrology, Institut d’Investigació en Ciències de la Salut Germans Trias i Pujol, Universitat Autònoma de Barcelona, Esfera UAB, Badalona, Spain; 3 Department of Pathology, Hospital Universitari Germans Trias i Pujol, Universitat Autònoma de Barcelona, Esfera UAB, Badalona, Spain; 4 Department of Clinical Biochemistry, Hospital Universitari Germans Trias i Pujol, Universitat Autònoma de Barcelona, Esfera UAB, Badalona, Spain; 5 Department of Medicine, Universitat Autònoma de Barcelona, Esfera UAB, Badalona, Spain; Institut national de la santé et de la recherche médicale (INSERM), France

## Abstract

**Background:**

Minimal change disease (MCD) and primary focal segmental glomerulosclerosis (FSGS) are the main causes of primary idiopathic nephrotic syndrome in children and adults, with diagnosis being essential for the appropriate choice of therapy and requiring renal biopsy. However, the presence of only normal glomeruli on renal biopsy of FSGS patients may lead to the misclassification of these patients as having MCD. The aim of this study was to (i) compare the peptide profile of MCD and FSGS patients with that of a group of healthy subjects, (ii) generate and validate a class prediction model to classify MCD and FSGS patients and (ii) identify candidate biomarkers of these glomerular entities by analysis of the urinary peptidome.

**Methods:**

The urinary peptide profile was analyzed by magnetic bead-based technology combined with MALDI-TOF mass spectrometry in 44 patients diagnosed of MCD (n = 22) and FSGS (n = 22). The resulting spectra were compiled and analyzed using ClinProTools software.

**Results:**

A class prediction model was developed to differentiate MCD and FSGS patients. The validation of this model correctly classified 81.8% (9/11) of MCD patients and 72.7% (8/11) of FSGS patients. Moreover, the signal with *m/z* 1913.60, identified as a fragment of uromodulin, and the signal with *m/z* 2392.54, identified as a fragment of alpha-1-antitrypsin, showed higher and lower peak areas, respectively, in FSGS patients compared with MCD patients.

**Conclusions:**

The simple, non-invasive technique described in the present study may be a useful tool to help clinicians by confirming diagnoses achieved by renal biopsy, thereby reducing misdiagnoses and avoiding the implementation of inappropriate therapies.

## Introduction

Chronic kidney disease is a public health problem worldwide with an increasing incidence and prevalence, poor outcome and high associated costs [1]. The common causes of chronic kidney disease are glomerular diseases, such as minimal change disease (MCD) and focal segmental glomerulosclerosis (FSGS), which are often associated with nephrotic syndrome in children and adults [2], [3].

Renal biopsy is needed to obtain the definitive diagnosis of glomerular diseases, to establish the prognosis, and to choose the most appropriate therapy. However, the invasiveness of this technique may result in complications and may be contraindicated in some cases [4], [5], [6]. Renal biopsy evaluation requires examination of the tissue under light, immunofluorescence, and electron microscopy, and an adequate sample size must be obtained, with a minimum number of glomeruli to demonstrate renal injury in cases of focal lesions [7], [8].

Light microscopy reveals apparently normal glomeruli in MCD and segmental sclerosis in some but not all glomeruli in FSGS. Accordingly, renal biopsies of FSGS patients showing only normal glomeruli may lead to the misclassification of these patients as MCD, especially in the earlier, pre-scarring stages of the disease.

Patients with MCD usually respond to corticosteroid therapy but a considerable number of patients with FSGS are dependent on or resistant to this treatment [9], [10]. Thus, the different treatment approaches and the toxicity of corticosteroids make it especially interesting to differentiate between these disorders.

Physiological and pathological processes may be reflected by peptides and proteins present in blood, urine and other body fluids. Proteins are differentially expressed as a consequence of the development of a disease and are, thus, very valuable as potential diagnostic biomarkers. In the case of kidney diseases, the urinary proteome has been extensively investigated [11], [12], [13], [14]. Urine is an ideal source of biomarkers because it can be obtained noninvasively, in large amounts and at minimum cost. Moreover, the protein and peptide content of urine is relatively homogeneous, probably because urine remains in the bladder for several hours and proteolytic degradation by endogenous proteases is completed before voiding [15].

In the last decade, mass spectrometry (MS) has been the method of choice for the analysis of peptides and small proteins in biological fluids. To reduce the complexity of biological samples prior to MS analysis, functionalized magnetic beads have been designed, which allow the capture and purification of peptides and small proteins and also allow the removal of salts to increase the sensitivity of the analysis. The combination of magnetic beads with matrix-assisted laser desorption/ionization time-of-flight (MALDI-TOF) MS has become a promising approach in the field of biomarker discovery and proteomic pattern diagnostic since it enables the rapid study of thousands of peptides and small proteins simultaneously with only a small sample volume and with high sensitivity. Moreover, the reproducibility of this approach may be improved by automation in a liquid-handling platform. This proteomic approach has been successfully used to profile the peptidome of different biological fluids [16], [17], [18], [19], [20], [21].

The objectives of our study were to (i) compare the peptide profile of MCD and FSGS patients with that of a group of healthy subjects, (ii) generate and validate a class prediction model able to classify MCD and FSGS patients, and (iii) identify potential biomarkers that discriminate between MCD and FSGS patients.

## Subjects and Methods

### Subjects and Sample Collection

This prospective study included Caucasian patients older than 18 years, with clinical signs of nephrotic syndrome, such as proteinuria, with stable renal function. Only patients with a clinical and histological diagnosis of MCD (n = 22) and primary FSGS (n = 22; 58% FSGS not otherwise specified, 14% perihiliar variant, 9% cellular variant, 14% tip variant, and 5% collapsing variant) were included. Cases of clinical or pathological features indicating a secondary cause such as autoimmune diseases, infections, cancer or exposure to nephrotoxic drugs were excluded.

Twenty-three of the 44 patients had also been studied in a previous report [12]. However, the urinary peptide profile of these patients was generated again to minimize intra-assay variations.

Urine and blood samples were collected the day of renal biopsy, prior to performing it.

Urine samples from 16 healthy subjects (10 females, 37±13 years) with normal renal function were collected to establish a normal urinary peptide profile.

The Clinical Research Ethics Committee of Germans Trias i Pujol Hospital approved the study protocol, and all patients gave written informed consent to participate.

### Renal Biopsy

Histological diagnosis was achieved by percutaneous renal biopsy performed before initiating corticosteroid or immunosuppressive therapy.

Biopsies were carried out using a Bard Monopty Disposable Core Biopsy Instrument (Bard Biopsy Systems, Tempe, AZ, USA) and processed for light, immunofluorescence, and electron microscopy following standard procedures. Light microscopy sections were stained with hematoxylin/eosin, Schiff’s periodic acid, methenamine silver, Masson’s trichrome and Congo red. Immunofluorescence assays were performed by incubating cryostat sections with polyclonal fluorescein isothiocyanate-conjugated secondary antibodies against IgG, IgM, IgA, C3 fraction, C1q, C4, kappa and lambda chains and fibrinogen (Dako Corporation, Copenhagen, Denmark).

### Study Design

MCD and FSGS patients were randomly subdivided into a preliminary training group for the generation of a class prediction model (11 MCD patients and 11 FSGS patients) and a validation group (11 MCD patients and 11 FSGS patients).

The spectral data obtained from the whole study population was also used for the identification of peptide signals differentially expressed among MCD patients, FSGS patients and healthy subjects.

### Biochemical Estimations

Biochemical variables were determined with a routine clinical chemistry laboratory analyzer immediately after extraction. Serum levels of total cholesterol and triglycerides were determined by conventional enzymatic methods.

Serum creatinine levels were determined using a modified Jaffe kinetic reaction (Roche Diagnostics, Basel, Switzerland). Twenty-four hour proteinuria was measured spectrophotometrically on a Cobas u 711 analyzer (Roche Diagnostics). The glomerular filtration rate was calculated using the Modification of Diet in Renal Disease (MDRD) formula.

### Peptidome Isolation

Urine samples were centrifuged at 2,100 g for 30 minutes at 4°C to remove cellular debris. The supernatant was recovered, adjusted to neutral pH with 1 M NH_4_HCO_3_, aliquoted, and immediately frozen at −80°C until processing.

Samples were thawed and pre-fractionated using Dynabeads RPC18 (Invitrogen, Breda, The Netherlands). Samples were processed in duplicate following the manufacturer’s protocol but modified for optimization purposes, as described previously [22]. Fifteen microliters of peptide eluate were obtained from each sample, diluted 1∶5 with LC-grade water (Lab-Scan, Gliwice, Poland), and mixed 1∶2 with matrix solution (1.84 mg/ml 2,6-dihidroxyacetophenone, 20% acetonitrile, 40 mmol/l ammonium citrate dibasic). Of the resulting mixture, 1µl was spotted in duplicate onto the sample anchor spots of an AnchorChip 600/384 target plate (Bruker Daltonics, Bremen, Germany) and allowed to air-dry at room temperature to let the matrix crystallize. Four spots of each sample were analyzed by MALDI-TOF MS. ClinProt Peptide Calibration Standard I (Bruker Daltonics), a commercially available mixture of protein/peptide calibrators, was mixed 1∶1 with matrix solution and 0.4 µl were deposited onto calibrant anchor spots of the AnchorChip target plate for instrument calibration.

### Robotics

Automation of the complete magnetic bead pre-fractionation and AnchorChip target plate loading was performed on a liquid-handling robotic platform (Freedom Evo, Tecan, Männedorf, Switzerland) to improve throughput and ensure assay reproducibility.

### MALDI-TOF MS

Mass spectrometry analyses were performed in an UltrafleXtreme MALDI-TOF/TOF mass spectrometer (Bruker Daltonics). Ionization was achieved by irradiation with a 337-nm nitrogen laser operating in linear positive ion mode geometry, with a repetition rate of 1,000 Hz. Each spectrum was acquired manually with 300 laser shots delivered randomly over the surface of the spot at a fixed laser power of 70%. Operating conditions were as follows: ion source voltages, 25 and 22.40 kV; reflector 1, 26.45 kV; reflector 2, 13.40 kV; pulsed ion extraction time, 300 ns. Spectra were externally calibrated, achieving a mass accuracy lower than 10 ppm. Peaks with a signal-to-noise ratio >3 in the *m/z* range of 1–10 kDa were recorded with the FlexControl acquisition software v3.4 (Bruker Daltonics).

### Bioinformatics

Due to the duplicates in pre-fractionation and in AnchorChip target plate loading, 4 spectra were obtained from each sample. A detailed analysis was performed with DataAnalysis software v3.4 (Bruker Daltonics) in order to choose the best spectrum for each sample. Spectra with the highest number of peaks and the highest intensity were selected. To assess the reproducibility of the magnetic bead-based technology used in this study, we analyzed the 4 replicated spectra obtained from 5 random samples. Six *m/z* signals were randomly selected to calculate the coefficient of variance of their peak area.

ClinProTools software v2.2 (Bruker Daltonics) was used to process MALDI-TOF MS spectra according to the following standard workflow: baseline subtraction to remove broad structures, normalization of spectra to their own total ion count, recalibration of spectra using the most prominent peaks, calculation of total average spectrum, peak area detection on the total average spectrum, and area calculation of each peak.

The generation of a class prediction model able to differentiate between MCD and FSGS patients was achieved with the Support Vector Machine (SVM) algorithm. Only spectra from the training groups were used, and the number of input peaks was automatically detected based on clustering of the peak rankings as determined by the SVM. The algorithm determines optimal separation planes between the different data classes. To determine the accuracy of the class prediction model the software offers values of cross validation and recognition capability. Cross validation is a measure for the reliability of a calculated model and can be used to predict how a model will behave in the future. This method is used for evaluating the performance of a classifier for a given data set and under a given parameterization. Recognition capability describes the performance of an algorithm, i.e., the proper classification of a given data set.

The model generated was further tested with spectral data from the validation groups.

For statistical analyses, peak area data provided by ClinProTools software was converted to ASCII files, exported to Excel spreadsheets and analyzed with SPSS software v15.0 (SPSS Inc., Chicago, Ill., USA).

### Protein Identification

Protein identification was conducted by HPLC-MS/MS as described in a previous report [12].

### Statistical Analysis

Continuous variables were expressed as medians (interquartile ranges). Differences between groups were tested by the non-parametric Mann-Whitney U test. Categorical variables were analyzed with the chi-square or Fisher’s exact probability test. Associations between biochemical variables and the peak area of the *m/z* signals were estimated using the Spearman’s correlation coefficient. Statistical analyses were performed with the SPSS software v15.0. A p-value <0.05 was considered statistically significant.

## Results

### Demographic and Clinical Characteristics

Demographic and clinical characteristics of glomerular patients, randomly divided into training or validation group, are presented in Table 1.

**Table 1:** Demographic and clinical characteristics of the study population.

	MCD			FSGS				
	Training	Validation	*P*_T-V_	Training	Validation	*P*_T-V_	*P*_T-T_	*P*_V-V_
No. of subjects	11	11		11	11			
Age (years)	38 (28–68)	68 (28–75)	0.374	57 (31–62)	55 (33–65)	0.718	0.358	0.490
Female/male ratio	4/7	3/8		3/8	4/7			
BMI (kg/m^2^)	24.2 (22.1–33.8)	26.4 (24.7–34.6)	0.497	25.9 (23.1–26.7)	25.3 (22.1–26.3)	0.288	0.778	0.223
TG (mg/dl)	230 (176–365)	163 (108–232)	0.070	239 (129–360)	114 (70–166)	0.056	0.923	0.266
TC (mg/dl)	362 (274–477)	278 (226–346)	0.151	217 (180–320)	197 (178–247)	0.602	0.076	0.071

Data are shown as median (interquartile range). Differences between groups were tested using the non-parametric Mann-Whitney U test. *P*_T-V_ shows P value between training and validation groups, *P*_T-T_ between training groups, and *P*_V-V_ between validation groups of both glomerular entities. A P value less than 0.05 was considered significant.

BMI: body mass index; TG: triglycerides; TC: total cholesterol.

Regarding age, no significant differences were found between patients in training and validation groups. There were no significant differences in patients’ age of both training groups.

**Figure 1:**
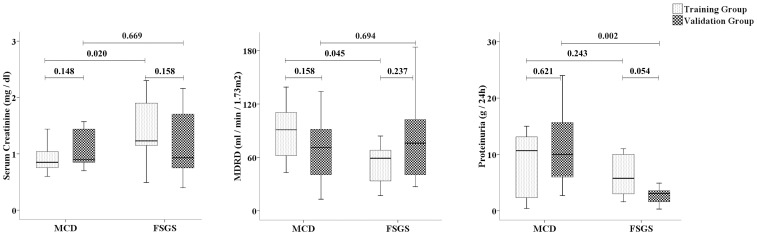
Box plots comparing the renal function of MCD and FSGS patients in the training and validation groups. a) Serum creatinine levels; b) MDRD formula and c) 24 h-proteinuria. The boxes indicate median and 25th and 75th percentiles. Data were compared using the Mann-Whitney U test. A P<0.05 was considered significant.

Differences in renal function were observed between patients of both training groups, with higher levels of serum creatinine in FSGS patients. No differences in proteinuria levels were found between these patients (Figure 1).

### Reproducibility of Urinary Peptide Profiling

The reproducibility of the technology used in this study was assessed by analyzing the spectral data from 5 randomly selected samples. The peak area of 6 *m/z* signals showed a mean coefficient of variance below 11% (Table 2).

**Table 2:** Reproducibility of urinary peptide profiling by magnetic-bead technology in combination with MALDI-TOF MS analysis.

			*m/z*						
			1798.56	1913.60	2392.54	2408.26	2642.26	2939.95	3004.65
		Rep 1	13.57	14.79	31.64	55.2	26.08	37.39	37.58
Urine	Peak Area	Rep 2	13.49	12.40	32.35	45.54	25.55	37.61	31.29
sample	(a.u.)	Rep 3	12.09	11.79	26.90	41.87	26.80	43.06	38.00
#1		Rep 4	15.36	15.88	32.95	48.49	25.98	36.93	34.00
	CV (%)		9.82	14.12	8.91	11.81	1.99	7.46	9.01
		Rep 1	21.07	294.10	20.88	24.04	14.01	19.71	46.64
Urine	Peak Area	Rep 2	26.71	235.66	22.32	26.24	12.68	20.46	49.63
sample	(a.u.)	Rep 3	20.87	270.95	23.06	23.33	13.30	19.79	50.17
#2		Rep 4	27.25	368.98	24.53	26.52	12.91	17.49	46.88
	CV (%)		14.50	19.29	6.69	6.35	4.41	6.67	3.78
		Rep 1	20.00	11.12	102.17	146.83	19.26	21.16	113.13
Urine	Peak Area	Rep 2	21.92	10.51	116.68	173.27	20.05	18.57	104.43
sample	(a.u.)	Rep 3	18.05	10.79	108.95	147.57	22.97	19.74	108.97
#3		Rep 4	19.82	11.21	100.57	145.21	22.85	23.88	109.32
	CV (%)		7.95	2.94	6.86	8.75	8.96	10.97	3.27
		Rep 1	13.29	126.69	20.90	49.75	17.56	17.26	101.82
Urine	Peak Area	Rep 2	12.17	131.38	21.87	50.44	17.10	15.02	113.95
sample	(a.u.)	Rep 3	15.21	138.46	21.23	46.74	17.45	17.33	88.43
#4		Rep 4	14.16	149.39	25.55	64.31	17.22	17.15	106.11
	CV (%)		9.43	7.23	9.59	14.83	1.22	6.69	10.42
		Rep 1	11.38	12.20	90.32	61.88	33.43	51.34	50.29
Urine	Peak Area	Rep 2	11.58	11.15	112.10	67.35	34.04	56.13	58.17
sample	(a.u.)	Rep 3	11.59	12.91	114.72	71.03	37.43	57.25	48.92
#5		Rep 4	10.47	11.18	105.45	64.47	41.61	62.21	62.77
	CV (%)		4.74	7.21	10.35	5.94	10.26	7.86	11.94
	Mean CV (%)		9.29	10.16	8.48	9.53	5.37	7.93	7.68

Peak area, in arbitrary units (a.u.), of the 7 peaks used for the generation of the class prediction model from 5 randomly selected urine samples processed in quadruplicate.

Rep: replicate; CV: coefficient of variance.

### Class Prediction Model

The spectra obtained from the training groups were analyzed by ClinProTools software to generate a class prediction model. Seven regions of the spectra, with *m/z* 1798.56, 1913.60, 2392.54, 2408.26, 2642.26, 2939.95 and 3004.65, were used for classification. The model allowed distinguishing between MCD and FSGS patients with a recognition capability of 100% and a cross validation of 55%.

### Validation of the Class Prediction Model

To verify the accuracy of the established classification model, the spectra from the validation groups were tested. The model correctly classified 81.8% (9/11) of samples from MCD patients and 72.7% (8/11) of samples from FSGS patients (Table 3).

**Table 3:** Evaluation of the class prediction model generated with ClinProTools software using spectral data from the validation groups.

	Validation groups	
	MCD (n = 11)	FSGS (n = 11)
Classified as MCD (n)	9	3
Classified as FSGS (n)	2	8
Correctly classified (%)	81.8	72.7

### Differentially Expressed Peptides between Glomerular Patients and Healthy Subjects

Urinary peptide profiles of glomerular patients differed significantly from those of healthy subjects (Table 4). Twenty-two signals discriminated MCD patients from healthy subjects; seven signals, with *m/z* 1769.38, 1898.37, 1913.60, 2713.96, 2976.97, 3004.65 and 3389.12, showed a higher peak area and 15 signals, with *m/z* 1945.50, 1961.71, 2305.01, 2378.07, 2392.54, 2408.26, 2491.41, 2505.64, 2521.45, 2543.26, 2642.26, 2939.95, 3161.72, 3226.48 and 4013.38, showed a lower peak area in healthy subjects.

**Table 4:** Peak area of urinary peptides of the study population.

				Healthy vs. MCD		Healthy vs. FSGS		MCD vs. FSGS				
m/z	Healthy subjects	MCD patients	FSGS patients	*P*	Fold(a)	*P*	Fold(b)	*P*	Fold(c)	Peptide sequence	Protein	Swiss-Prot accession number
1769.38	41.2 (27.6–81.0)	17.7 (9.0–41.2)	42.2 (15.8–96.2)	0.017	1.3	0.806		0.099				
1831.61	9.2 (5.9–15.8)	12.4 (9.2–17.0)	14.8 (8.3–112.0)	0.071		0.030	0.1	0.512		LVRYTKKVPQVSTPTL	ALB	P02768
1898.37	77.4 (59.8–139.6)	19.4 (12.0–29.6)	21.4 (10.9–34.8)	<0.001	4.1	<0.001	3.0	0.808		SVIDQSRVLNLGPITRK	UMOD	P07911
1913.60	378.2 (346.1–644.6)	18.0 (10.1–45.8)	41.0 (14.2–138.8)	<0.001	15.4	<0.001	5.5	0.039	0.4	SGSVIDQSRVLNLGPITR	UMOD	P07911
1945.50	7.9 (6.0–11.1)	56.6 (17.9–170.3)	50.4 (31.9–170.9)	<0.001	0.1	<0.001	0.1	0.942		EAIPMSIPPEVKFNKPF	A1AT	P01009
1961.71	11.0 (8.0–14.9)	21.1 (9.9–67.8)	14.2 (11.7–66.1)	0.021	0.2	0.037	0.4	0.610		EAIPM_ox_SIPPEVKFNKPF	A1AT	P01009
2305.01	6.4 (5.3–8.7)	12.3 (8.7–20.3)	10.7 (8.9–20.2)	0.005	0.3	0.001	0.1	0.884				
2378.07	16.8 (15.2–19.7)	39.5 (27.4–146.4)	29.7 (18.6–61.7)	<0.001	0.1	0.005	0.2	0.080				
2392.54	13.4 (12.0–16.9)	217.4 (83.1–319–4)	95.5 (28.1–194.8)	<0.001	0.1	<0.001	0.1	0.024	1.7	MIEQNTKSPLFMGKVVNPTQK	A1AT	P01009
2408.26	12.5 (10.2–15.9)	58.1 (41.1–119.1)	37.7 (17.8–55.4)	<0.001	0.2	<0.001	0.3	0.016	1.8			
2491.41	12.4 (8.6–18.5)	53.8 (25.2–110.7)	25.6 (18.5–65.5)	<0.001	0.2	0.001	0.3	0.109				
2505.64	5.6 (4.5–14.8)	99.3 (47.5–201.4)	94.0 (26.9–161.9)	<0.001	0.1	<0.001	0.1	0.451		LMIEQNTKSPLFMGKVVNPTQK	A1AT	P01009
2521.45	8.3 (6.7–10.5)	35.1 (16.9–62.8)	25.7 (12.5–50.3)	<0.001	0.2	<0.001	0.2	0.343		LMIEQNTKSPLFM_ox_GKVVNPTQK	A1AT	P01009
2543.26	8.9 (7.1–12.3)	20.8 (15.2–30.4)	23.1 (18.0–70.5)	0.000	0.5	<0.001	0.2	0.159				
2642.26	5.8 (4.9–7.5)	16.3 (7.4–27.6)	18.2 (11.7–143.5)	0.001	0.3	<0.001	0.1	0.132				
2678.13	12.6 (10.3–19.4)	22.0 (14.3–33.3)	21.6 (14.8–27.7)	0.076		0.037	0.4	0.923				
2713.96	55.5 (38.9–77.5)	35.0 (23.8–53.1)	28.3 (17.8–45.6)	0.029	1.4	<0.001	2.0	0.224		LLKNGERIEKVEHSDLSFSKDWS	B2M	P61769
2939.95	16.9 (14.7–21.8)	23.2 (18.2–58.0)	20.0 (13.5–71.3)	0.007	0.2	0.232		0.593				
2976.97	60.4 (44.1–70.5)	38.4 (15.7–55.0)	14.4 (8.8–30.4)	0.005	1.7	<0.001	3.4	0.008	2.0			
3004.65	118.8 (73.2–166.7)	49.6 (16.1–110.3)	31.4 (12.4–96.0)	0.011	1.7	0.001	2.4	0.285				
3161.72	19.9 (16.3–26.6)	52.9 (18.4–87.5)	20.6 (9.2–39.6)	0.009	0.3	0.806		0.011	2.8			
3226.48	14.8 (11.0–17.3)	19.8 (16.1–28.2)	18.7 (15.2–46.1)	0.002	0.6	0.023	0.3	0.752				
3389.12	119.1 (93.2–165.0)	42.0 (17.7–58.0)	23.3 (9.0–50.9)	<0.001	2.0	<0.001	3.8	0.072				
4013.38	45.5 (30.8–54.3)	70.7 (48.1–130.3)	55.5 (23.9–98.7)	0.010	0.4	0.270		0.253				

Data are expressed in arbitrary units as median (interquartile ranges). The nonparametric Mann Whitney U test was performed to assess differences.

(a) Ratio between the median values of the peak area in healthy subjects and MCD patients.

(b) Ratio between the median values of the peak area in healthy subjects and FSGS patients.

(c) Ratio between the median values of the peak area in MCD and FSGS patients.

ALB: albumin; UMOD: uromodulin; A1AT: alpha-1-antitrypsin; and B2M: beta-2-microglobulin.

Twenty signals showed statistically different area (Table 4) on comparing healthy subjects with FSGS patients; six signals, with *m/z* 1898.37, 1913.60, 2713.96, 2976.97, 3004.65 and 3389.12, showed a higher peak area and 14 signals, with *m/z* 1831.61, 1945.50, 1961.71, 2305.01, 2378.07, 2392.54, 2408.26, 2491.41, 2505.64, 2521.45, 2543.26, 2642.26, 2678.13 and 3226.48, showed a lower peak area in healthy subjects.

### Differentially Expressed Peptides between MCD and FSGS Patients

Statistically significant differences in 5 signals were observed on comparing the peptide profile of MCD and FSGS patients (Table 4). One signal, with *m/z* 1913.60, showed a higher peak area and 4 signals, with *m/z* 2392.54, 2408.26, 2976.97 and 3161.72, showed a lower peak area in FSGS patients compared with MCD patients.

### Peptide Identifications

**Figure 2:**
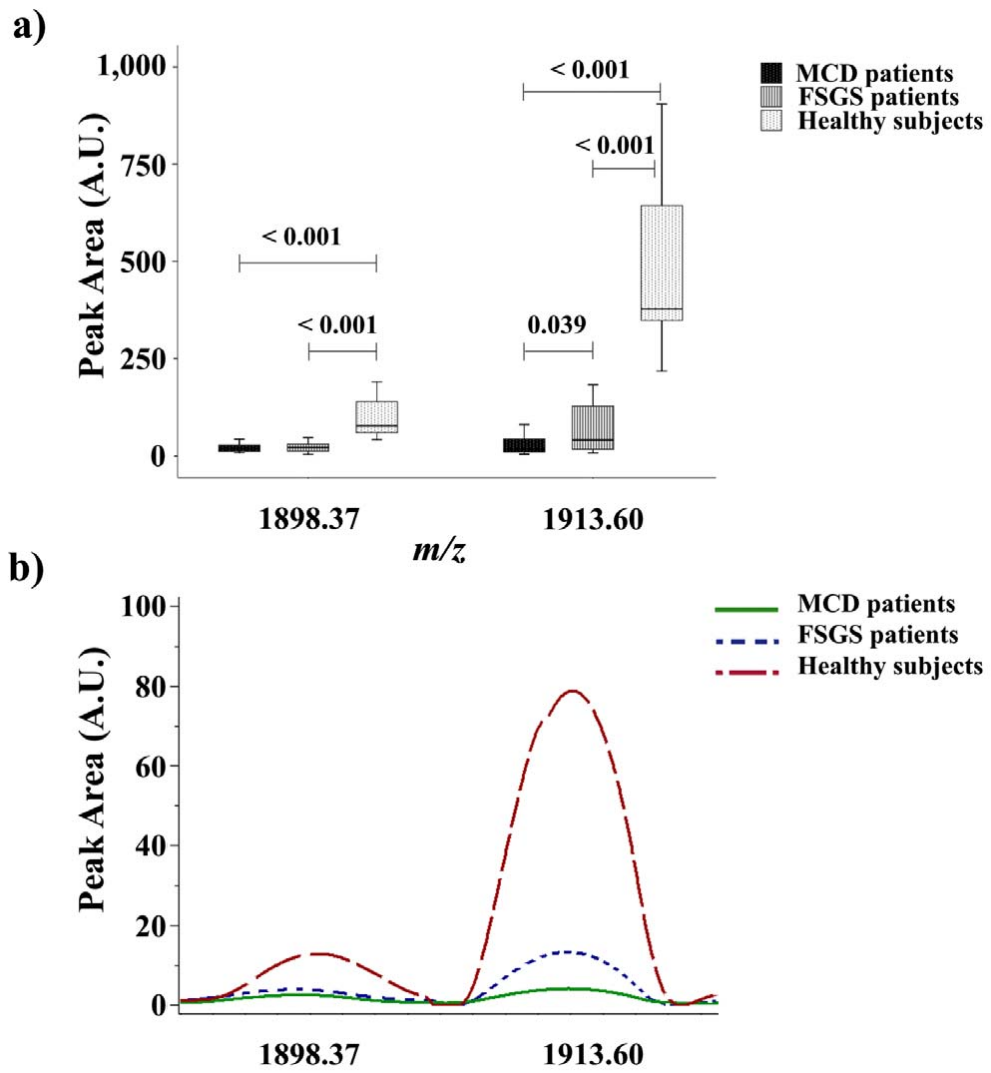
Uromodulin peptides (*m/z* 1898.37 and 1913.60) in urine from glomerular disease patients and healthy subjects. a) Box plot showing urinary expression of uromodulin peptides in MCD, FSGS patients and healthy subjects. b) ClinProTools image showing the average intensity, in arbitrary units, of uromodulin peptides in MCD, FSGS patients and healthy subjects.

Signals with *m/z* 1898.37 and 1913.60 have been identified in previous reports as fragments of uromodulin (UMOD; Swiss-Prot accession No.: P07911; *Homo sapiens*; the amino acid sequences matched were 591–607 and 589–606, respectively) [12]. These signals showed a higher peak area in healthy subjects compared with MCD and FSGS patients. FSGS patients showed a higher peak area in signal *m/z* 1913.60 than MCD patients (Figure 2).

**Figure 3:**
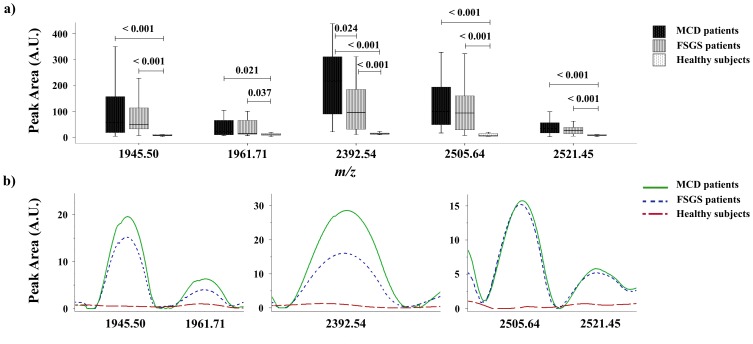
Alpha-1-antitrypsin peptides (*m/z* 1945.50, 1961.71, 2392.54, 2505.64 and 2521.45) in urine from glomerular disease patients and healthy subjects. a) Box plot showing urinary expression of alpha-1-antitrypsin peptides in MCD, FSGS patients and healthy subjects. b) ClinProTools image showing the average intensity, in arbitrary units, of alpha-1-antitrypsin peptides in MCD, FSGS patients and healthy subjects.

Signals with *m/z* 1945.50, 1961.71, 2392.54, 2505.64 and 2521.45 have been identified in previous reports as fragments of alpha-1-antitrypsin (A1AT; Swiss-Prot accession No.: P; *Homo sapiens*; the amino acid sequences matched were 378–394, 378–394, 398–418, 397–418, and 397–418, respectively) [12]. These signals showed a lower peak area in healthy subjects compared with MCD and FSGS patients. A lower peak area in signal with *m/z* 2392.54 was observed in FSGS patients on comparing with MCD patients (Figure 3).

Signal with *m/z* 2713.96, identified as a fragment of beta-2-microglobulin (B2M; Swiss-Prot accession No.: P61769; *Homo sapiens*; the amino acid sequence matched was 59–81) [12], showed a higher peak area in healthy subjects compared with MCD and FSGS patients.

Signal with *m/z* 1831.61, identified as a fragment of serum albumin protein (ALB; Swiss-Prot accession No.: P02768; *Homo sapiens*; the amino acid sequence matched was 432–447) [12], showed a lower peak area in healthy subjects compared with FSGS patients, but no differences were found on comparing healthy subjects with MCD patients.

We analyzed correlations of signals corresponding to fragments of UMOD, A1AT, B2M and ALB with patients’ age, gender, serum creatinine, MDRD and proteinuria levels to determine whether any clinical parameter influenced these results, but no association was observed.

## Discussion

Our results revealed differences in the urinary peptide profile on comparing glomerular patients with healthy subjects. Furthermore, a class prediction model able to classify MCD and FSGS patients was generated.

During the last decade, proteomic studies based on magnetic bead technology combined with MS readout have demonstrated the utility of this approach in profiling the low-molecular-weight proteome of different biological fluids [16], [17], [23], [24]. The reproducibility of this type of profiling within and between days has been evaluated [25], [26]. In our study, a standardized protocol for sample collection and processing, including the use of a robotic platform, was strictly followed to minimize variations. Indeed, the intra-assay imprecision achieved was less than 11%, indicating that the results obtained with this methodology are highly reproducible for peptidome profiling of human urine.

Urinary proteomics has been widely performed to identify biomarkers of clinical diseases, mainly of those affecting the kidney [11], [12], [13], [14], [27], [28]. Within the group of kidney diseases, those affecting the glomerulus have been studied and potential biomarkers have been proposed, but none has been confirmed to discriminate between the different entities [28], [29]. In a previous study we identified differentially expressed urinary peptides which allowed distinguishing between glomerular kidney disease patients and healthy subjects [12]. We therefore endeavored to search for non-invasive biomarkers able to differentiate FSGS and MCD patients because of the need for specific treatment in each disease and diagnosis by renal biopsy may be confounded if the sample does not include the affected portion of the kidney. Consequently, in the present study we included patients diagnosed with MCD or FSGS in order to develop a class prediction model able to differentiate between these entities.

The model generated correctly classified 81.8% and 72.7% of MCD and FSGS patients from validation groups, respectively.

In addition to creating the classification model, we considered it to be of interest to compare the urinary peptidome of patients of both glomerular entities with the aim of determining the presence of differentially expressed peptides between them. Identification of the proteins to which these peptides belong could help to gain more insight into the pathological mechanisms involved in these diseases.

Interestingly, although 18 urinary peptides showed a similar peak area on comparing MCD and FSGS patients, some differences were observed in other peptides; FSGS patients showed a higher peak area in one signal corresponding to a fragment of UMOD and a lower peak area in one signal corresponding to a fragment of A1AT. Our results also showed that healthy subjects had higher areas in signals of UMOD peptides and lower areas in signals of A1AT peptides compared to patients with glomerular diseases. The area of these signals was independent of the degree of proteinuria, thus offering additional information in the diagnosis of these diseases, since proteinuria alone is not enough to differentiate between glomerular entities.

UMOD, also known as the Tamm-Horsfall protein, is the most abundant urinary protein in healthy individuals; it is synthesized exclusively in the kidney, on the epithelial cells of the thick ascending limb (TAL) of Henle’s loop [30]. Although the physiological role of this protein remains unclear, recent studies have proposed low levels of UMOD as a biomarker of renal disease [14], [31]. In this regard, our results are in agreement. The peak area of the signal *m/z* 1913.60, corresponding to a peptide of UMOD, showed a higher peak area in healthy subjects and also allowed differentiation between MCD and FSGS patients, with higher values in the latter; underexpression of this UMOD peptide has been previously described in patients with advanced renal disease and diabetic patients and may be due to an alteration of the apical membrane of the TAL epithelial cells [32], [33].

A1AT is a major protease inhibitor in human serum that inhibits neutrophil elastase [34]. Its deficiency is associated with lung, liver and skin disease [35], [36]. Associations between this glycoprotein and vascular disease, inflammatory bowel disease, vasculitis and glomerulonephritis have been proposed, albeit not definitively established. In a previous study, Candiano *et al.* analyzed the urinary proteome from patients with primary nephrotic syndrome by combining two-dimensional electrophoresis with MS, and found fragments of albumin and A1AT as the most abundant proteins. Although these fragments were mainly formed in plasma, a few were produced in urine, suggesting the presence in urine of specific proteases [29]. In agreement with this study, we found 5 peptides corresponding to A1AT with higher intensities in glomerular patients. Interestingly, one of these peptides allowed differentiation between MCD and FSGS patients. Moreover, with regard to albumin fragments, we found a peptide signal (*m/z* 1831.61) with a higher peak area in FSGS patients compared with healthy subjects.

The fragmentation pattern of the proteins described above may reflect the proteolytic activity that takes place during kidney disease, and the appearance of specific peptides in the urine could consequently serve as biomarkers of the diseases studied here, MCD and FSGS.

Histological diagnosis takes time and may not achieve a precise diagnosis if an adequate tissue sample is not obtained. Consequently, renal biopsy is limited, time-consuming and cannot be performed several times, thereby limiting its practice in the follow-up of the patients. The search for biomarkers in urine could replace renal biopsy as an accurate, non-invasive test and could be repeated to follow the progression of the disease and monitor the response to therapy.

Although great efforts are being made in the search for biomarkers able to distinguish between MCD and FSGS, and some candidates have been proposed [37], to our knowledge, none has yet been confirmed. Our study suggests that analysis of urinary UMOD and A1AT peptides may present a non-invasive method for distinguishing between these two glomerular entities. However, these proteins are candidate biomarkers that must be tested in assays with a larger number of patients. In addition, the remaining peaks found in this study should also be identified since this might provide new insight into the pathological processes that occur in these diseases.

In conclusion, given the difficulty in differentiating between MCD and FSGS by evaluation of renal biopsies in some cases, corroboration with a simple and non-invasive technique, such as that described here, could help clinicians to confirm the diagnosis and thereby avoid unnecessary or inadequate treatments.
